# University of Buffalo. Commencement

**Published:** 1852-03

**Authors:** 


*University of Buffalo. Commencement.*—The Annual Commencement Exercises in the Med. Department of the University of Buffalo, took place on the 25th ult., at the American Hall, and were of an unusually interesting character.

After a prayer by the Rev. Dr. Thomson, an eloquent address was delivered, and degrees conferred upon twenty graduates by his Honor, James Wadsworth, Mayor of the city, and *ex-officio* member of the Council of the University.

Prof. C. A. Lee followed by a charge to the graduates, in which the trials and joys of the life of the physician were depicted in an admirable manner, coupled with parting counsel so impressively given, that it will not be likely to be forgotten.

The benediction was pronounced by the Rev. Mr. Ingersoll.

The music was furnished by the Buffalo Academy of Instrumental Music, and added much to the pleasure of the occasion.

The honorary degree of Doctor in Medicine, was conferred upon Charles H. Wilcox, of Buffalo, and Walter Elliot Lauderdale, of Geneseo, Livingston county, N. Y.

The following is a list of the names of those upon whom the degree of Doctor of Medicine was conferred in course:—

Lyman Congdon, Venice, Cayuga co., N. Y.; Elias R. Weaver, Groton, Tompkins co., N. Y.; Edward P. Christian, Detroit, Mich.; Robert H. Dee,

Stamford, Canada West; Joseph A. Burrows, Newark, Wayne co., N. Y.; John C. Crooks, Allen Hill, Ontario co., N. Y.; David B. Van Slyke, Cicero, Onondaga co., N. Y.; George H. Briggs, Nunda, Livingston co., N. Y.; George Abbott, Buffalo, Erie co., N. Y.; George W. Whiting, Chautauque, Chautauque co., N. Y.; John R. Cotes, Batavia, Genesee co., N. Y.; John D. Dunning, Webster, Monroe co., N. Y.; Ernst Godfrey Pupikofer, Switzerland; Charles S. Hurlbut, Olean, Cattaraugus co., N. Y.; James W. Chadwick, Victoria, Norfolk co., C. W.; James B. Colgrove, Sardinia, Erie co., N. Y.; Hiram Taber, Bergen, Genesee co., N. Y.; Edwin H. Gibbs, Buffalo, Erie co., N. Y.; James M. Selfridge, Waterloo, Seneca co., N. Y.; Alfred M. Burnham, Pavillion, Genesee co., N. Y.

